# Dietary Walnuts Protect Against Obesity-Driven Intestinal Stem Cell Decline and Tumorigenesis

**DOI:** 10.3389/fnut.2018.00037

**Published:** 2018-05-31

**Authors:** Fangxia Guan, Tahmineh Tabrizian, Ardijana Novaj, Masako Nakanishi, Daniel W. Rosenberg, Derek M. Huffman

**Affiliations:** ^1^Department of Molecular Pharmacology, Albert Einstein College of Medicine, Bronx, NY, United States; ^2^Department of Medicine, Albert Einstein College of Medicine, Bronx, NY, United States; ^3^School of Life Sciences, Zhengzhou University, Zhengzhou, China; ^4^Institute for Aging Research, Albert Einstein College of Medicine, Bronx, NY, United States; ^5^School of Medicine, University of Connecticut Health, Farmington, CT, United States

**Keywords:** colon cancer, obesity, walnuts, inflammation, intestine

## Abstract

Obesity can negatively impact intestinal homeostasis, and increase colon cancer risk and related mortality. Thus, given the alarmingly high rates of obesity in the US and globally, it is critical to identify practical strategies that can break the obesity-cancer link. Walnuts have been increasingly recognized to mitigate cancer risk, and contain many bioactive constituents with antioxidant and anti-inflammatory properties that could potentially counteract pathways thought to be initiators of obesity-related cancer. Therefore, the purpose of this study was to determine if walnuts could preserve intestinal homeostasis, and attenuate tumorigenesis and growth in the context of obesity and a high calorie diet. To this end, we studied effects of walnuts on these parameters under different dietary conditions in wildtype mice, two independent *Apc* models (*Apc*^1638N/+^ and *Apc*^Δ14^), and in MC38 colon cancer cells *in vivo*, respectively. Walnuts did not alter the metabolic phenotype or intestinal morphology in normal mice fed either a low-fat diet (LFD), LFD with 6% walnuts (LFD+W), high-fat diet (HFD), or HFD with 7.6% walnuts (HFD+W). However, walnuts did lead to a significant reduction in circulating CCL5 and preserved intestinal stem cell (ISC) function under HFD-fed conditions. Furthermore, walnuts reduced tumor multiplicity in *Apc*^1638N/+^ male HFD+W animals, as compared to HFD controls (3.7 ± 0.5 vs. 2.5 ± 0.3; *P* = 0.015), tended to reduce the number of adenocarcinomas (0.67 ± 0.16 vs. 0.29 ± 0.12; *P* = 0.07), and preferentially limited tumor growth in *Apc*^Δ14^ male mice (*P* = 0.019) fed a high-calorie western-style diet. In summary, these data demonstrate that walnuts confer significant protection against intestinal tumorigenesis and growth and preserve ISC function in the context of a high-calorie diet and obesity. Thus, these data add to the accumulating evidence connecting walnuts as a potentially effective dietary strategy to break the obesity-colon cancer link.

## Introduction

Obesity is a major risk factor for colon cancer, as well as accelerated disease progression (1) and related mortality (2, 3). Indeed, a meta-analysis of prospective studies determined that an elevated body mass index (BMI>30) increases the risk for this disease by 30-70% in men, but less so in women (4). While general obesity (BMI) is clearly linked to colon cancer risk, the distribution of body fat appears to be even more revealing, as surrogate measures of abdominal obesity more strongly predict colon cancer risk in both men and women (5), and we have shown that this link is causal in a mouse model (6). While the role of excess weight on cancer risk is apparent, the hazards posed by obesity on cancer mortality are even more striking, and is nearly universal among the most common forms of cancer, including an increased risk of death from colon cancer by 46% in women and by nearly two-fold in men (2, 7).

Recently, it has become clear that the ramifications of obesity on intestinal homeostasis can be observed well before the onset of tumor formation, with marked effects on tissue morphology (8), function (9), and a “molecular signature” which may “prime” intestinal cells toward a more tumorigenic predisposition (10), increasing tumor incidence and progression in the presence of an oncogenic insult. Indeed, we and others have shown a marked increase in tumor incidence within the context of obesity, utilizing either *Apc* mutant mice (6) or azoxymethane (AOM) treatment (11, 12), to initiate tumor development in rodents. Likewise, pre-clinical models have demonstrated that obesity accelerates tumor growth and metastatic disease. Given the alarmingly high rates of obesity in the US and globally, and its clear link to cancer, it is critical to identify practical strategies that can break this link.

Walnuts (*Juglans regia*), which are well known to promote cardiovascular health, have also been reported to reduce risk for type 2 diabetes, cognitive decline, and all-cause mortality (13–16). Indeed, walnuts contain many bioactive constituents, including omega-3 fats (i.e., ALA), ⋎-tocopherol, phytosterols and several polyphenolic compounds that harbor anti-oxidant and anti-inflammatory properties (17). More recently, a growing number of reports have documented cancer protective effects of walnuts and its components in cells as well as in animal models of prostate, breast and colon cancer (18–25). Indeed, early studies in colon cancer cells documented cytotoxic and pro-apoptotic effects of individual walnut components, while an initial study in mice demonstrated that a walnut-supplemented diet significantly slowed the growth of HT-29 human colon cancer cells *in vivo* (17). These effects were attributed to a suppression of angiogenesis and alterations in the miRNA expression and fatty acid composition of tumor cells (26, 27). More recently, walnut consumption was demonstrated to reduce tumor burden in an AOM model of colon cancer selectively in male mice (24). Interestingly, walnut consumption was also linked to alterations in the microbial community structure associated with reduced tumor progression (24), which was corroborated by a study in Fischer 344 rats, which demonstrated increased abundance of *Firmicutes* with reduced abundance of *Bacteriodetes* (28).

As obesity has been shown to accelerate intestinal tumor formation and progression (6, 9, 29–32), practical dietary strategies centered on natural foods to mitigate this risk are warranted. Given the documented beneficial effects of walnuts on the gut, coupled with its potential to counteract many risk factors thought to define the obesity-cancer link, the purpose of this study was to understand to what extent, if any, walnuts harbor the ability to preserve intestinal homeostasis, and attenuate intestinal tumorigenesis and growth in the context of obesity and a high calorie diet.

## Materials and methods

### Experimental diets

Fresh shelled walnuts were provided by the California Walnut Commission. For diet production, walnuts were shipped to Envigo (formerly Harlan, Madison, WI) where they were ground on site and incorporated into appropriate experimental diets and soft-vacuum packed into 1 kg bags to preserve freshness. A total of four pelleted diets were generated for this study: a control low-fat diet (LFD), an isocaloric LFD in which walnuts were incorporated at 6% by weight (LFD+W), a control high fat diet (HFD), and an isocaloric HFD containing 7.67% walnuts by weight (HFD+W). The walnut content for the HFD was adjusted to account for the anticipated reduction in food intake for mice consuming more energy dense food. The detailed diet formulations are shown in Table 1. Walnut doses were chosen based upon prior studies in rodents which have found these amounts to be most beneficial for cancer prevention (24) and attenuation of behavioral deficits in Alzheimer's Disease models (33). Diets were routinely replaced with fresh pellets twice per week and individual bags were only used for up to 2 weeks after breaking the vacuum seal. In addition, a total western diet (TWD) containing either 0% or 7% walnuts was also utilized for a parallel study in *Apc*^Δ14^ mice (24). All experimental animals were housed and treated in accordance with protocols reviewed and approved by the Institute for Animal Care and Use Committee at the Albert Einstein College of Medicine (Protocol #20150103) and the Center for Comparative Medicine (CCM) at UConn Health (Protocol #101369-0519), respectively.

**Table 1:** Dietary formulations of control and walnut-supplemented low fat and high fat diets.

**Component (g/kg)**	**LFD**	**LFD+W**	**HFD**	**HFD+W**
Casein	210	200	245	231.75
Sucrose/Corn Starch	90/465	90/455	200/85	200/85
Maltodextrin	100	100	115	102
Walnuts, %	0	6	0	7.6
Lard and Soybean Oil	20/20	0.45/0.45	195/30	146/30
Kcal from CHO, %	69.1	69.1	36.0	36.0
Kcal from Protein, %	20.5	20.5	19.0	19.0
Kcal from Fat, %	10.4	10.4	45.0	45.0
Kcal/g	3.6	3.6	4.6	4.6

### Experiment 1 design: intestinal phenotyping

Three week-old male C57BL/6J mice were obtained from The Jackson Labs (Bar Harbor, ME) for general phenotyping and intestinal characterization studies. Animals were housed in the animal facility at Einstein, kept under standard conditions and provided *ad libitum* access to food and water. After 1 week of acclimatization, mice were single housed and assigned to either LFD, LFD+W, HFD or HFD+W (*n* = 12 per group). Body weight was monitored weekly and food intake was measured twice per week until 24 weeks of age. Body composition was evaluated at 22 weeks of age by quantitative magnetic resonance (qMR Echo MRI). After 20 weeks on diet, insulin sensitivity was evaluated by insulin tolerance tests (ITT) at a dose of 1 mU/kg insulin by intraperitoneal (i.p.) injection in random fed mice. At study completion, animals were fasted 4 h and sacrificed for collection of plasma and tissues for biochemical assays. In addition, the gastrointestinal tract was carefully and rapidly excised and staged as a Swiss roll for paraffin embedding and histopathologic analysis.

### Experiment 2 design: intestinal tumorigenesis in *Apc*^1638N/+^ mice

*Apc*^1638N/+^ male mice on a C57BL/6 background and female C57BL/6J mice (Jackson Labs, Bar Harbor, ME) were mated to generate *Apc*^1638N/+^ male offspring. We have previously used this mutant model to demonstrate that obesity and specifically visceral fat, is implicated in tumor development of these mice (6). This model also has the benefit of a significant latency in pathology, with tumor initiation beginning around 4 months of age and most animals demonstrating tumor burden by 6–8 months of age. At 4 weeks of age, *Apc*^1638N/+^ male mice were assigned to either LFD (*n* = 11), LFD+W (*n* = 12), HFD (*n* = 18) or HFD+W groups (*n* = 23 group). Animals assigned to LFD groups were then followed for up to 32 weeks of age and animals assigned to the HFD groups were followed for up to 24 weeks of age. A power analysis, based upon our prior study (6), determined a sample size of *n* = 18 per group would be necessary to detect a 25% reduction in tumor multiplicity with 80% statistical power. While these numbers were achieved in the HFD study, the number of LFD animals in which pathologic data was collected at the study endpoint was underpowered in relation to our target sample size, due to some premature mortality in these groups. At necropsy, the entire intestine was quickly excised, surrounding mesenteric fat removed, and the gut divided into duodenum, jejunum, ileum and colon, as previously described (6). Each segment was opened longitudinally, rinsed in ice-cold phosphate-buffered saline, and carefully flattened for examination of tumor multiplicity with the aid of a dissecting, magnifying lens. Adenomas (~ >0.5 mm diameter) when present, were counted in each segment of intestinal tissue and recorded.

### Experiment 3 design: intestinal tumorigenesis in *Apc*^Δ14^ mice

Beginning at 4 weeks of age, *Apc*^Δ14^ mice were fed the TWD (Harlan Laboratories) supplemented with 0% or 7% walnuts by weight, and the composition of these diet formulations have been described previously (24). Male (Con TWD *n* = 12, TWD+7% Walnut *n* = 11) and female (Con TWD *n* = 10, TWD+7% Walnut *n* = 9) mice were provided the experimental diets for 12 weeks and then animals were sacrificed and intestinal tissue excised and processed as described earlier (24), for assessment of tumor incidence and growth throughout the gastrointestinal tract.

### Experiment 4 design: colon cancer cell progression

In order to determine whether walnuts can protect against more advanced disease, 8 week-old male C57BL/6J mice (*n* = 48) were obtained from The Jackson Labs and group housed 4 per cage. Following a 1 week acclimation, animals were assigned to either LFD, LFD+W, HFD or HFD+W diets (*n* = 12 group) for approximately 12 weeks. Tumor progression was then evaluated using an established tumor inoculation protocol by injection of MC38 cells (a kind gift of Shoshana Yakar) into the right rear flank (0.5 × 10^6^ MC38 cells) of syngeneic C57BL/6J mice. Tumor volume was then monitored over 19 days with calipers and calculated by the equation V = L × W^2^ × 0.5, where L is length and W is width of the tumor (34), and tumor weights were recorded at sacrifice. A tumor length and width measuring in excess of 1.25 cm, evidence of ulceration at the tumor sight, or a body condition score <2 were considered humane endpoints during the study. Some animals met or exceeded the tumor size criteria by day 19 and the study was immediately terminated at this point. MC38 cells were maintained in Dulbecco's Modified Eagle Medium (DMEM) containing 1 mM sodium pyruvate, 2 mM L-glutamine and 1 mM non-essential amino acid and supplemented with 10% fetal bovine serum (FBS) (GIBCO, USA), at 37°C in the presence of 5% CO_2_. Obesity was previously shown to increase growth and proliferation of MC38 cells following injection (35), establishing this model as an ideal tool to test for effect of walnuts on cancer progression.

### Morphometric analysis

Evaluation of the gastrointestinal tract was performed following isolation and division into four segments: duodenum, jejunum, ileum and colon, as previously described (6, 36). Tissues were rolled and fixed overnight in 10% neutral-buffered formalin at 4°C, processed through a series of alcohols and xylenes, and embedded in paraffin. Hematoxylin & Eosin (H&E) stained sections (5 μm) from each segment of small intestine were then scanned into digital files with a PerkinElmer P250 High Capacity Slide Scanner, and crypt area and villi length were evaluated in Panoramic Viewer using 50 random villi per animal to form a single composite average.

### Western blotting

Western blotting was performed as described (37). Protein was extracted from frozen tissues in RIPA buffer and total protein content was determined using the BCA protein assay (Sigma, St. Louis, Mo) with BSA as a standard. Proteins were separated by SDS-PAGE, transferred onto PVDF membranes and incubated with an appropriate primary and secondary antibody. Equal loading and transfer was confirmed by staining, imaging and quantifying the original gel, using Biorad stain-free gel technology. Immunoblotting was performed for pS6 (#5364), total S6 (#2217), pAkt ^Ser473^ (#4060), total Akt (#4691), p-p44/42MAPK^Thr202/Tyr204^ (#9101) total p44/42 MAPK (#4695), and β-catenin (#8480), all from Cell Signaling. Bands were visualized by chemiluminescence to first indication of pixel saturation using a Biorad Chemidoc bioimager and densitometry performed using Image Lab 4.1 (Biorad, Hercules, CA).

### Gene expression in colon and small intestine

Total RNA from frozen tissues was isolated using the Trizol procedure as described previously (36). In brief, first-strand complementary DNA (cDNA) was synthesized with random primers and total RNA as a template using Biorad iScript cDNA Synthesis Kit. All qPCR reactions were carried out using Biorad Sso Advanced SYBR Green mix on a Biorad CFX384 qRT-PCR Machine. All data were then normalized to the housekeeping gene, 18S, using the delta delta Ct method. Primer sequences are provided in Supplementary Table 1.

### Plasma measures

Whole blood was collected following a 4 h fast into K2-EDTA collection tubes (Sarstedt AG & Co; Numbrect, Germany), and plasma was separated from red blood cells by centrifugation (1,500 × g, 4°C, 15 min). Plasma free fatty acids (Wako Diagnostics, Richmond, VA, USA) and triglycerides (Sigma, St. Louis, MO) were analyzed using standard calorimetric assays. Plasma insulin levels were measured by a rat/mouse ELISA (EMD Millipore, Inc.) with rat insulin standards using a spectrophotometer (Biorad iMark platereader) following the manufacturer's instructions (36, 37). In addition, a Bio-Plex MAGPIX Multiplex Reader (Biorad Inc., Hercules, CA) was used to measure the following 23 inflammatory mediators simultaneously in plasma: Eotaxin, G-CSF, GM-CSF, IFN-γ, IL-10, IL-12 (p40), IL-12 (p70), IL-13, IL-15, IL-17, IL-1α, IL-1β, IL-2, IL-3, IL-4, IL-5, IL-6, IL-7, IL-9, IP-10, KC, LIF, LIX, M-CSF, MCP-1, MIG, MIP-1α, MIP-1β, MIP-2, RANTES, TNF-α, and VEGF.

### Adipose tissue cytokine array

Protein was extracted from epididymal fat in PBS buffer with Triton X-100 and protease inhibitors (Roche). Total protein content was determined using the BCA protein assay as above (Sigma, St. Louis, Mo) with BSA as a standard. 250 ug of total protein was applied to the membrane array and protein levels of 40 cytokines/chemokines were quantified according to the manufacturer's instructions (R&D Systems, Inc., Minneapolis, MN). Specific factors measured in this assay included: CXCL13, C5a, G-CSF, GM-CSF,I-309, Eotaxin, sICAM-1, IFN-γ, IL-1α, IL-1β, IL-1ra, IL-2, IL-3, IL-4, IL-5, IL-6, IL-7, IL-10, IL-13, IL-12p70, IL-16, IL-17, IL-23, IL-27, IP-10, I-TAC, KC, M-CSF, JE, MCP-5, MIG, MIP-1α, MIP-1β, MIP-2, RANTES, SDF-1,TARC, TIMP-1, TNF-α, TREM-1.

### Intestinal permeability

To determine intestinal barrier function, animals were fasted for 4 h and then gavaged (*n* = 10/group) and evaluated with the well-described 4,000 Da fluorescent dextran–FITC assay (DX-4000–FITC; Sigma-Aldrich, St. Louis, Missouri, USA) (38–40). Specifically, mice were fasted for a minimum of 4 h and then dosed with DX-4000–FITC by gavage (500 mg/kg body weight, 125 mg/ml). After 4 h, ~50 uL of blood was collected via tail bleed and plasma isolated. For measurement, plasma was diluted 1:1 in PBS (pH 7.4) and analyzed for FITC with a fluorescence reader (485 nm excitation and 535 nm) against a standard curve as described (41).

### Intestinal organoid assay

In order to assess intestinal stem cell (ISC) function, crypts were isolated from the small intestine of LFD, HFD and HFD+W fed mice (*n* = 4 group) following 4 weeks on diet, as previously described (36, 42). Isolated crypts were washed with ADF medium, centrifuged at 800 rpm for 5 min, re-suspended in ADF medium and counted on a hemocytometer. Approximately 250 crypts were then re-suspended in 25 uL of matrigel, transferred to a 48-well plate to solidify at 37°C for 30 min, and overlaid with 250 ul crypt culture medium (ADF 1x, Pen/Strep 1x, HEPES 1x, Glutamax 1x, N2 1x, B27 1x, N-acetyl-L-cysteine 1μM, Noggin 100 ng/ml, EGF 50 ng/ml, Rock inhibitor 10 μM, and R-Spondin 500 ng/ml) and maintained at 37°C. Fresh medium was applied every 3 days and the number and organoid formation on day 9 was assessed with a light microscope and normalized to the beginning number of counted crypts and expressed as organoids per crypt.

### Statistics

Parametric cross-sectional data were log- transformed to ensure normality of distribution and analyzed by either independent samples t-test, one-way or two-way ANOVA (diet x walnut) with Tukey *post-hoc* adjustment when appropriate, while longitudinal measurements were assessed by repeated measures ANOVA, using SPSS v16 (SPSS, Chicago, IL). For non-parametric data, or when normality could not be achieved, data were analyzed by the Kruskal-Wallis procedure, and *post-hoc* comparisons performed using the Mann-Whitney *U*-test. *P* ≤ 0.05 was considered significant.

## Results

### Dietary walnuts do not significantly affect the metabolic phenotype

**Figure 1:**
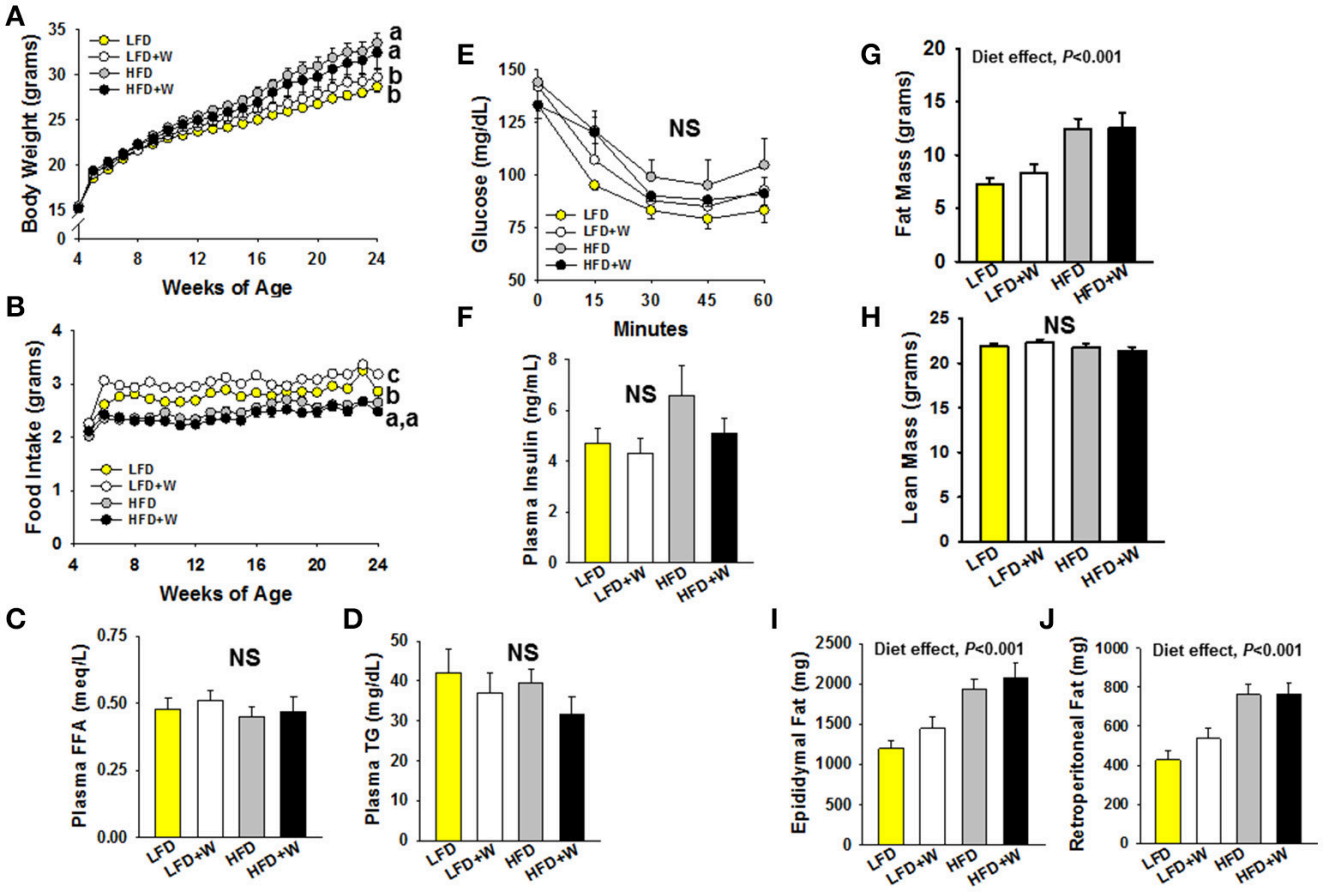
Effect of diet and walnut consumption on the metabolic phenotype in male C57BL/6J mice. **(A)** Mice fed a HFD were significantly heavier than LFD-fed counterparts, irrespective of walnut intake. **(B)** Mice consuming the LFD+W demonstrated a significant increase in food intake as compared to controls, while no difference was observed between HFD and HFD+W mice. **(C,D)** No significant effects of diet or walnut intake was observed for plasma FFA or TG levels (*n* = 12 per group) **(E,F)** Results of an ITT suggest no significant effects on insulin sensitivity, though HFD mice tended to be more resistant and hyperinsulinemic than other groups (*n* = 11–12 per group). **(G–J)** HFD groups were fatter than LFD-fed animals, including an increase in visceral fat, without effects of walnuts, while lean mass was similar across groups. Bars represent mean ± SE. NS, Not significant. Different letters denote a significant difference between groups, *P* ≤ 0.05.

We first set out to characterize the phenotypic effects of walnuts on the metabolic phenotype when exposed to LFD and HFD conditions. As shown in Figure 1A, HFD-fed mice were heavier than LFD-fed animals, and weight gain was not mitigated by walnuts under either condition. LFD+W mice consumed more food than LFD controls (Figure 1B; *P* < 0.05), but intake was similar between HFD and HFD+W groups (Figure 1B). However, FFAs and TGs were not significantly different among groups (Figures 1C,D). We next performed ITTs in random-fed mice (1 mU/kg dose) and also evaluated insulin levels after a 4 h fast. Overall, no significant difference in insulin sensitivity (Figure 1E) or insulin levels (Figure 1F) were observed among groups, though HFD mice tended to have higher insulin levels and more impaired insulin sensitivity than other groups. HFD *per se* also increased adiposity (Figure 1G), without impacting lean mass (Figure 1H), including a significant increase in visceral fat (Figures 1I,J; Diet effect = *P* < 0.001), and these effects were not moderated by walnuts.

### Walnuts preserve intestinal stem cell function under HFD conditions

**Figure 2:**
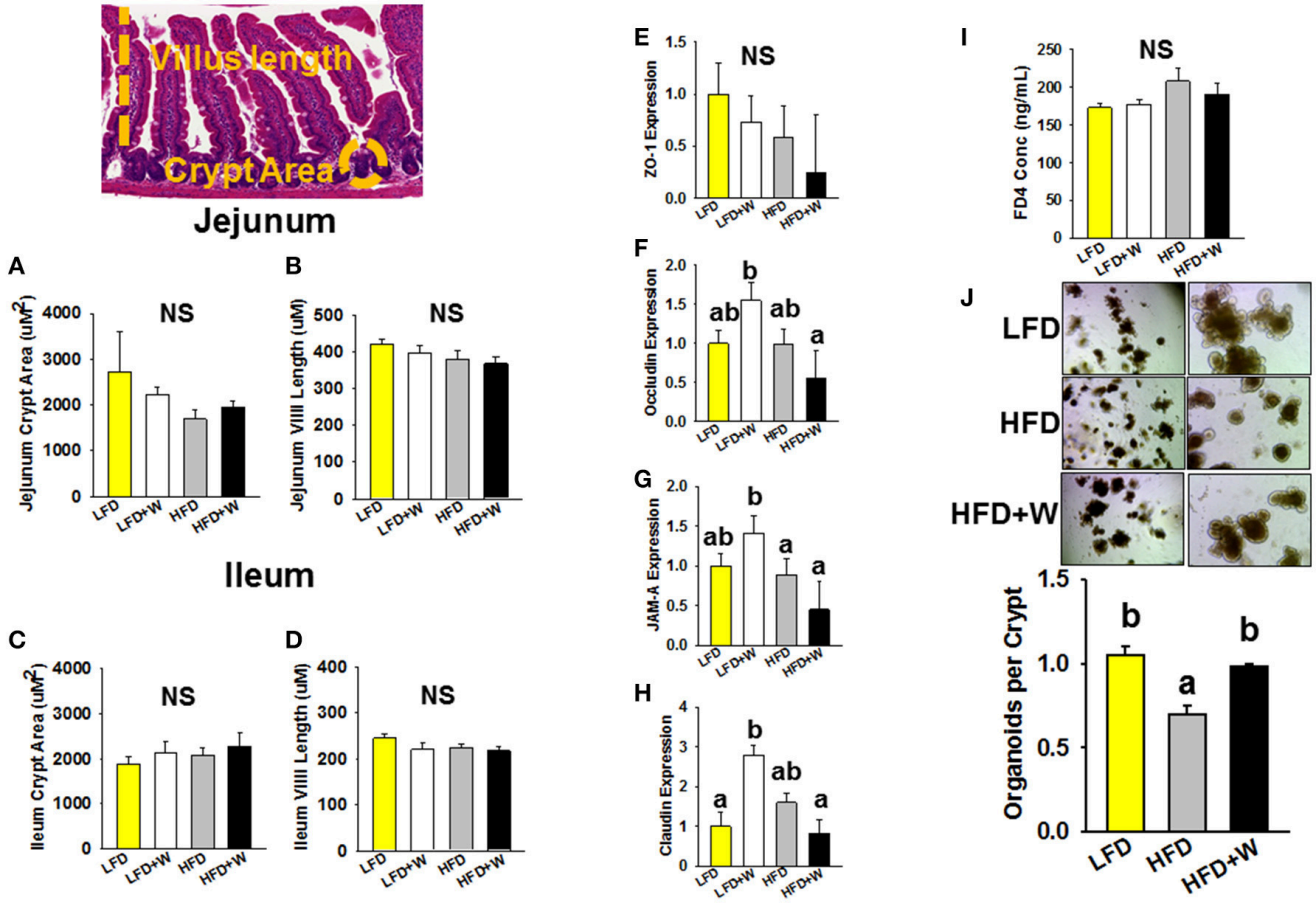
Effect of diet and walnut consumption on features of intestinal status and function**. (A–D)** Morphometric analysis revealed no significant difference in crypt area or villus length in jejunum or ileum (*n* = 9–12 per group). **(E–H)** Gene expression of intestinal tight junction-related genes in jejunum suggest that walnuts supplemented in the LFD upregulated occludin, JAM-A and claudin expression (*n* = 10 per group). **(I)** Measurement of intestinal leak *in vivo* using the FD4 assay revealed no significant difference in intestinal permeability among groups (*n* = 10 per group). **(J)** ISC function, as determined by an *ex vivo* organoid formation assay at day 9, revealed a significant impairment in organoid generation with HFD, but this reduction was prevented by inclusion of walnuts (*n* = 3 per group). Bars represent mean ± SE. NS, Not significant. Different letters denote a significant difference between groups, *P* ≤ 0.05.

We next examined the role of walnuts in the context of LFD and HFD conditions, on intestinal morphology, mucosal barrier integrity and ISC function. In both jejunum and ileum, we observed no effect of HFD or walnut intake on crypt area or villi length (Figures 2A–D). Interestingly, tight junction-related genes in jejunum were upregulated in LFD+W mice, with exception of ZO-1, but not in HFD+W animals (Figures 2E–H), while no differences were observed in colon (Supplementary Figure 1). In addition, we assessed intestinal membrane permeability *in vivo*, as determined by the appearance of FD4 in plasma (Figure 2I). In contrast to prior reports of increased intestinal “leak” with HFD, we observed only a marginal increase in plasma FITC from HFD-fed animals, and this parameter was not significantly altered by walnuts. Finally, we employed an *ex vivo* 3D intestinal organoid assay, previously used to demonstrate increased ISC proliferation by caloric restriction and rapamycin (42), and reduced function with aging. Following 4 weeks on a HFD, we observed a significant reduction in the ability of ISCs to generate organoid-like structures (Figure 2J). However, this decline in ISC function with HFD was completely prevented by walnuts (*P* < 0.05).

### Walnuts reduce systemic but not tissue levels of inflammation

**Figure 3:**
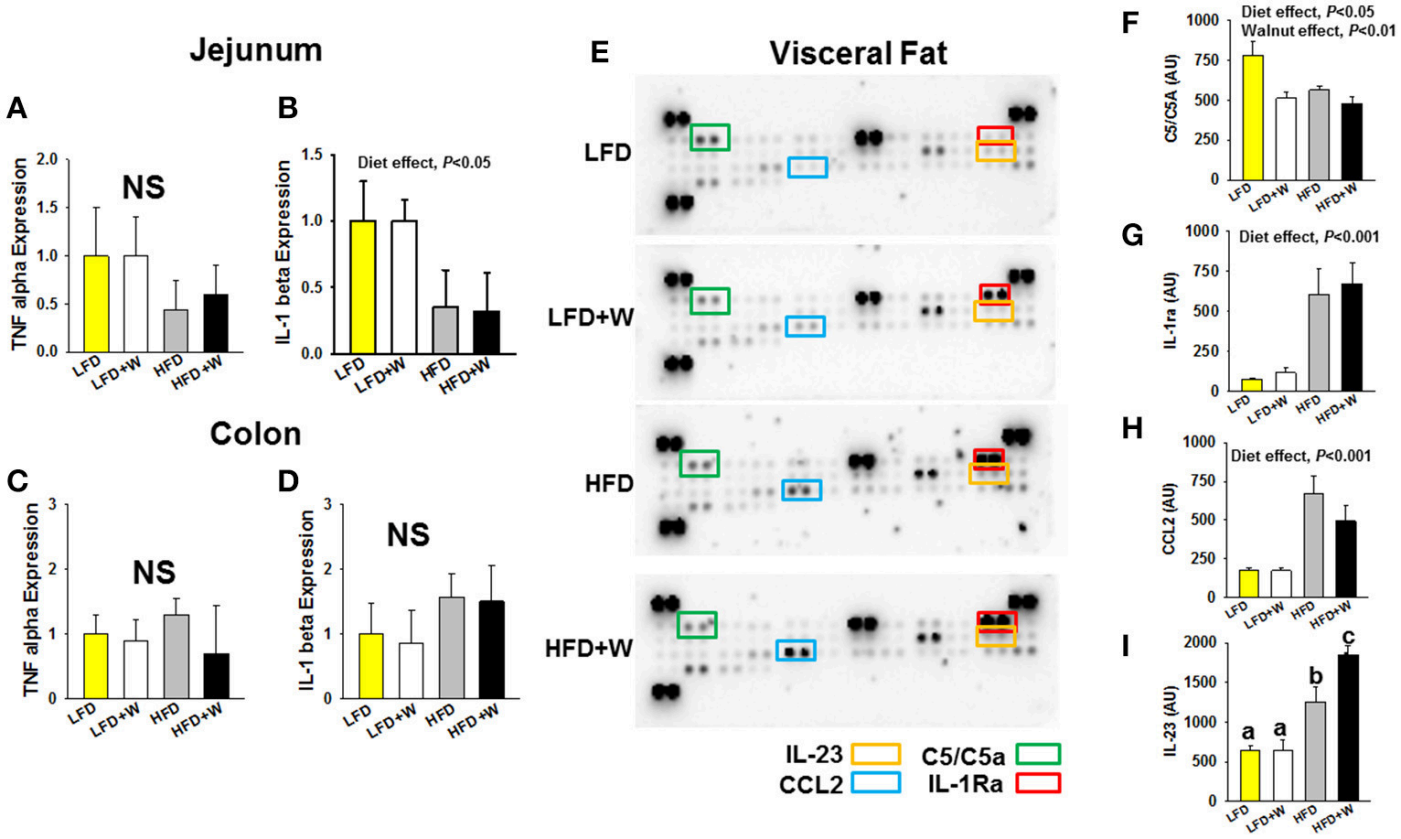
Effect of diet and walnut consumption on intestinal and adipose tissue inflammation. **(A–D)** Gene expression for TNFα and IL-1β in jejunum and colon failed to detect a significant change in inflammatory gene expression with HFD feeding or an effect of walnuts. To the contrary, a main effect of diet was observed in jejunum, indicating a reduction in cytokine expression by HFD *per se*, irrespective of walnuts (*n* = 10 per group). **(E–I)** Using a cytokine array in adipose tissue, a significant reduction in C5/C5a was observed with walnut diets, while IL-1ra and CCL2 were increased in both HFD and HFD+W groups (*n* = 4 per group). On the other hand, IL-23 levels were markedly elevated only in the HFD+W group. Bars represent mean ± SE. NS, Not significant. Different letters denote a significant difference between groups, *P* ≤ 0.05.

In order to characterize the inflammatory status of experimental animals, we first profiled plasma levels of cytokines and chemokines using a multiplex format. Among 23 analytes profiled, MIP-1α was reduced in both HFD groups, while only CCL5 was reduced in HFD+W mice, as compared to all other groups (Table 2; *P* < 0.05). At the tissue level, expression of TNFα in jejunum tended to be decreased with HFD or HFD+W feeding, which was significant for IL-1β (Figures 3A,B; Diet effect = *P* < 0.05), while no differences were observed in colon (Figures 3C,D). Meanwhile, a protein array in visceral adipose tissue determined that walnuts and HFD *per se* downregulated C5 levels, while other inflammatory markers were up-regulated with high-fat feeding, including IL-1ra, and CCL2 (Diet effect = *P* < 0.001), whereas IL-23 was increased only in HFD+W mice (*P* < 0.05), suggesting that walnuts did not interfere with adipose-related inflammation in obesity (Figures 3E–I).

**Table 2:** Inflammatory cytokines and chemokines in male C57BL/6J mice provided either a control or walnut-supplemented low fat or high fat diet.

**Analyte (pg/mL)**	**LFD (*n* = 9)**	**LFD+W (*n* = 10)**	**HFD (*n* = 10)**	**HFD+W (*n* = 10)**
IL-1α	44.9 ± 5.1	40.0 ± 6.5	67.1 ± 33.5	27.0 ± 5.2
IL-1β	562.5 ± 60.4	436.1 ± 60.2	403.9 ± 59.1	479.5 ± 119.1
IL-2	56.8 ± 10.7	49.0 ± 8.9	56.8 ± 10.7	34.1 ± 9.1
IL-3	19.6 ± 3.6	15.7 ± 3.9	13.4 ± 3.4	9.0 ± 3.7
IL-4	9.2 ± 3.1	12.3 ± 3.1	8.5 ± 2.3	12.8 ± 4.5
IL-5	40.2 ± 5.1	30.0 ± 6.7	25.8 ± 6.1	34.9 ± 8.8
IL-6	22.4 ± 3.8	14.5 ± 2.0	11.8 ± 3.0	12.7 ± 2.7
IL-9	ND	ND	ND	ND
IL-10	125.1 ± 15.2	86.2 ± 22.1	80.1 ± 20.1	53.4 ± 17.2
IL-12 (p40)	461.0 ± 126.4	341.4 ± 13.6	269.4 ± 19.8	282.3 ± 29.5
IL-12 (p70)	220.1 ± 30.2	167.0 ± 41.6	205.6 ± 52.1	106.3 ± 32.0
IL-13	309.6 ± 65.9	263.8 ± 53.8	218.5 ± 53.8	166.8 ± 56.4
IL-17	66.0 ± 13.5	55.8 ± 18.6	81.3 ± 25.4	32.6 ± 10.1
CCL-11	1898.9 ± 171.7	1447.0 ± 230.8	1253.4 ± 264.1	922.9 ± 234.8
G-CSF	79.7 ± 5.7	67.2 ± 5.4	92.1 ± 11.8	87.9 ± 18.3
GM-CSF	310.8 ± 41.7	238.6 ± 45.6	227.2 ± 45.9	144.5 ± 50.0
CXCL-1	84.4 ± 13.4	64.0 ± 5.9	61.4 ± 5.6	70.8 ± 13.4
MCP-1	474.0 ± 69.7	397.9 ± 74.4	331.8 ± 86.2	267.0 ± 73.7
MIP-1α	31.4 ± 1.8^a^	32.3 ± 2.8^ab^	24.9 ± 2.4^b^	23.7 ± 3.8^b^
MIP-1β	112.3 ± 15.0	82.2 ± 19.2	70.3 ± 20.6	52.1 ± 17.4
CCL5	48.1 ± 3.5^a^	46.9 ± 7.0^a^	40.3 ± 4.9^a^	23.1 ± 3.9^b^
IFN⋎	81.9 ± 11.5	81.2 ± 17.4	62.5 ± 17.2	37.7 ± 10.6
TNF-α	600.8 ± 79.8	488.5 ± 107.5	434.1 ± 118.9	324.0 ± 91.0

*Values are means ± SE. Plasma samples obtained from Experiment 1 were subjected to a multiplex assay and data were analyzed by the Kruskal-Wallis procedure and the Mann-Whitney U test when appropriate. Any value below the lower limit of detection of the assay was replaced by the minimal detectable concentration MOD for the specific analyte, and these values were ranked as a tie for purposes of the statistical analysis. Different letters denote a significant difference between groups for MIP-1a and CCL5 respectively, P ≤ 0.05*.

### Walnuts tend to preserve gut metabolic signaling while reducing β-catenin levels

**Figure 4:**
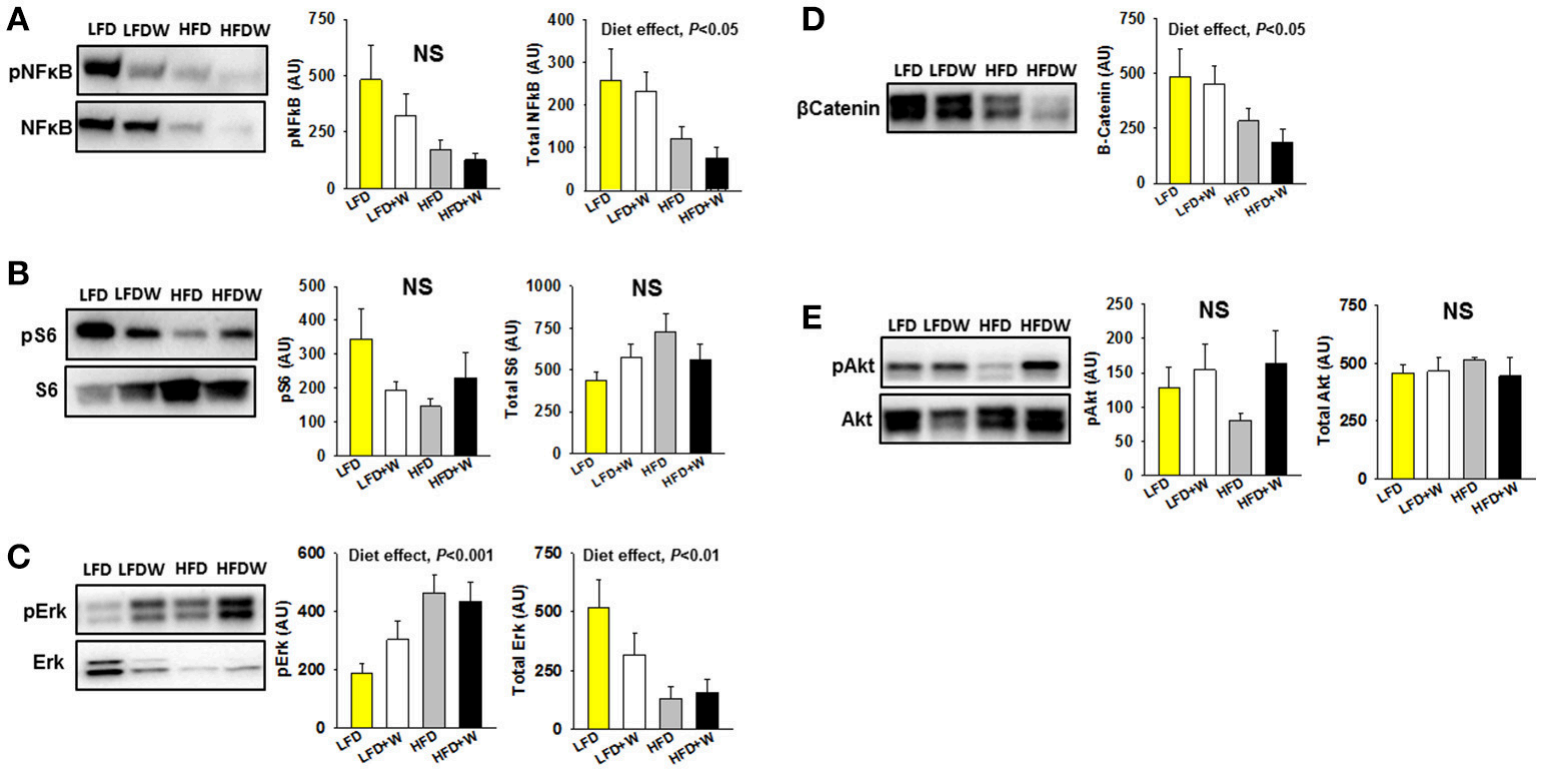
Effect of diet and walnut consumption on intestinal metabolic signaling pathways**. (A)** In jejunum, we observed a tendency for reduced pNFκB (p65) in HFD and HFD+W animals, while a significant diet effect was observed for total NFκB (*n* = 8 per group). **(B)** Both walnuts and HFD tended to reduce pS6, which sits downstream of various growth factor signaling pathways and the mTOR pathway. **(C,D)** High-fat feeding significantly increased pErk, with a concominant reduction in total Erk and β-catenin. **(E)** While not significant, pAkt levels tended to be reduced in HFD mice and better maintained in HFD+W animals. Bars represent mean ± SE. NS, Not significant. There were no signficant diet x walnut interactions among groups.

We next assessed metabolic signaling pathways related to intestinal homeostasis, including NF-κB, Akt and β-catenin. In jejunum, we observed that high-fat feeding per se significantly reduced total NF-κB (Figure 4A; Diet effect = *P* < 0.05), but not pS6 (Figure 4B), and significantly increased pErk (Diet effect = *P* < 0.001), with a concomitant reduction in total Erk (Diet effect = *P* < 0.01) and β catenin (Figures 4C,D; Diet effect = *P* < 0.05). Meanwhile, pAkt levels, which were numerically reduced in HFD mice, tended to be maintained in the HFD+W animals, but this did not reach significance (Figure 4E). In contrast, most metabolic signaling measures in colon were unaffected by HFD or walnuts, with the exception of a significant reduction in pErk in the HFD group, which was completely prevented by walnuts (Supplementary Figure 1; *P* < 0.05).

### Walnuts protect against obesity-driven intestinal tumorigenesis, but not progression

**Figure 5:**
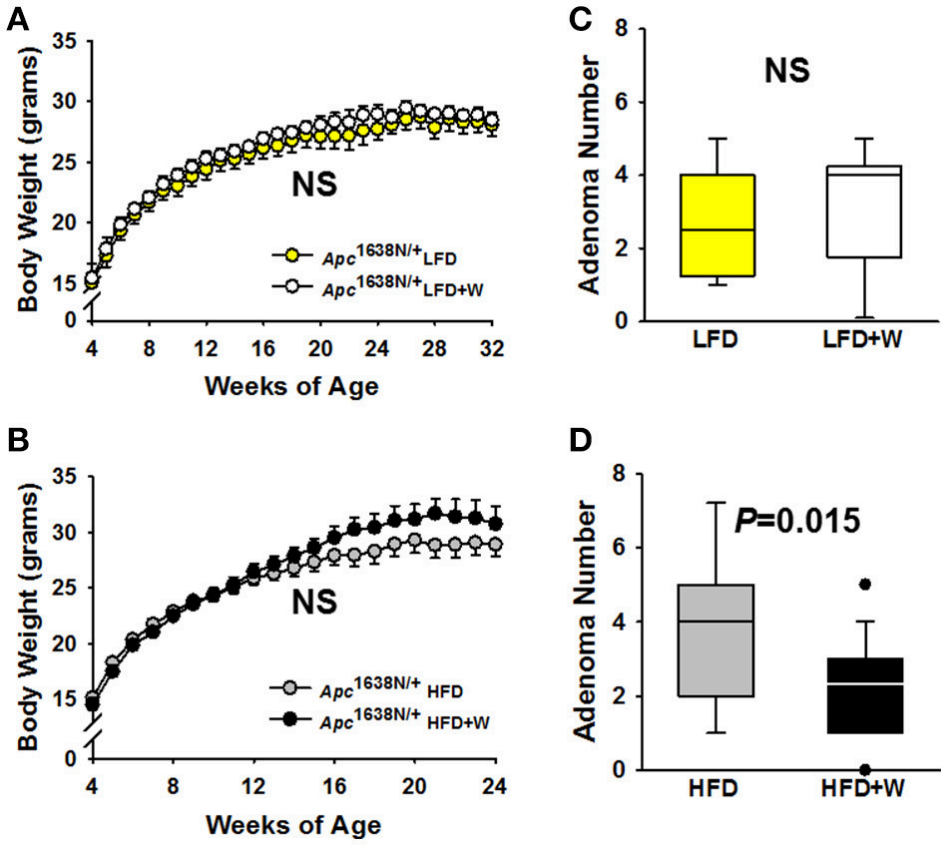
Effect of diet and walnut consumption on intestinal tumorigenesis in *Apc*^1638N/+^ male mice. Tumorigenesis was evaluated in *Apc*^1638N/+^ male mice on a control or walnut-supplemented LFD for up to 32 weeks of age [LFD (*n* = 11), LFD+W (*n* = 12)], while a second cohort was provided either a control or walnut-supplemented HFD for up to 24 weeks of age [HFD (*n* = 18), HFD+W groups (*n*=23 group)]. **(A,B)** Similar to results in wild type mice, walnuts did not alter weight gain on the LFD or HFD, respectively, over the course of the study. **(C)** At 32 weeks of age, post-mortem analysis revealed no significant differences in small intestinal tumor multiplicity, as determined by the number of adenomas between LFD and LFD+W mice. **(D**) In contrast to the LFD experiment, tumor numbers were significantly reduced in HFD+W animals, as compared to HFD controls (*P* = 0.015), which is supported by histopathologic analysis shown in Table 3. Lines and symbols represent mean ± SE. Box plots indicate median values and first to third quartile range, while lines indicate minimum and maximum range; symbols indicate outliers. NS, Not significant.

Consistent with observations in wild type mice, no difference in body weight was observed over 28 weeks of study (32 weeks old) in *Apc*^1638N/+^ male mice fed either a LFD (Figure 5A) or 20 weeks of study (24 weeks old) in the HFD-fed groups (Figure 5B), respectively, regardless of walnut supplementation. Furthermore, no significant difference was observed in small intestinal adenoma formation between LFD or LFD+W fed mice (Figure 5C; *P* = 0.71), though it should be acknowledged that the sample size of these groups were somewhat limited in power, as compared to the HFD study. On the other hand, HFD-fed mice demonstrated accelerated tumor development and increased multiplicity, as compared to LFD cohorts, necessitating the truncation of the study to 24 weeks in these mice. Remarkably, walnuts potently mitigated tumor formation in HFD-fed *Apc*^1638N/+^ males, reducing tumor burden by approximately 32% (Figure 5D; 3.7 ± 0.5 vs. 2.5 ± 0.3; *P* = 0.01). We further performed histopathology on intestinal tissues from the HFD cohort and observed a tendency toward reduced tubulovillous adenomas (*P* = 0.14) and adenocarcinomas (*P* = 0.07) in HFD+W mice (Table 3).

**Table 3:** Histopathology of the gastrointestinal tract in *Apc*^1638N/+^ mice provided either a control or walnut-supplemented high fat diet.

	**HFD (*n* = 18)**	**HFD+W (*n* = 16)**	***P*-value**
Hyperplasia, crypt epithelial, Focal^#^	1.16 ± 0.35	1.08 ± 0.34	0.87
Hyperplasia, crypt epithelial, Multifocal^#^	1.22 ± 0.11	1.17 ± 0.12	0.77
Dysplastic foci	0.39 ± 0.14	0.52 ± 0.21	0.63
Adenoma, tubular	0.61 ± 0.18	0.53 ± 0.21	0.77
Adenoma, tubulovillous	0.33 ± 0.11	0.12 ± 0.08	0.14
Total Adenoma	0.94 ± 0.21	0.64 ± 0.22	0.32
Adenocarcinoma, tubular	0.33 ± 0.14	0.11 ± 0.08	0.19
Adenocarcinoma, tubulovillous	0.33 ± 0.14	0.18 ± 0.10	0.37
Total Adenocarcinoma	0.67 ± 0.16	0.29 ± 0.12	0.07

Values are means ± SE. No differences were observed between HFD and HFD+W mice on crypt hyperplasia or occurrence of disyplastic foci. Consistent with an observed decrease in adenoma formation by gross examination, HFD+W mice tended to have a reduction in tubulovillous adenoma formation, and a trend for reduced adenocarcinoma development, P = 0.07.

# *Value based on the pathologic severity using a 1–4 scale, with 4 being most severe*.

**Figure 6:**
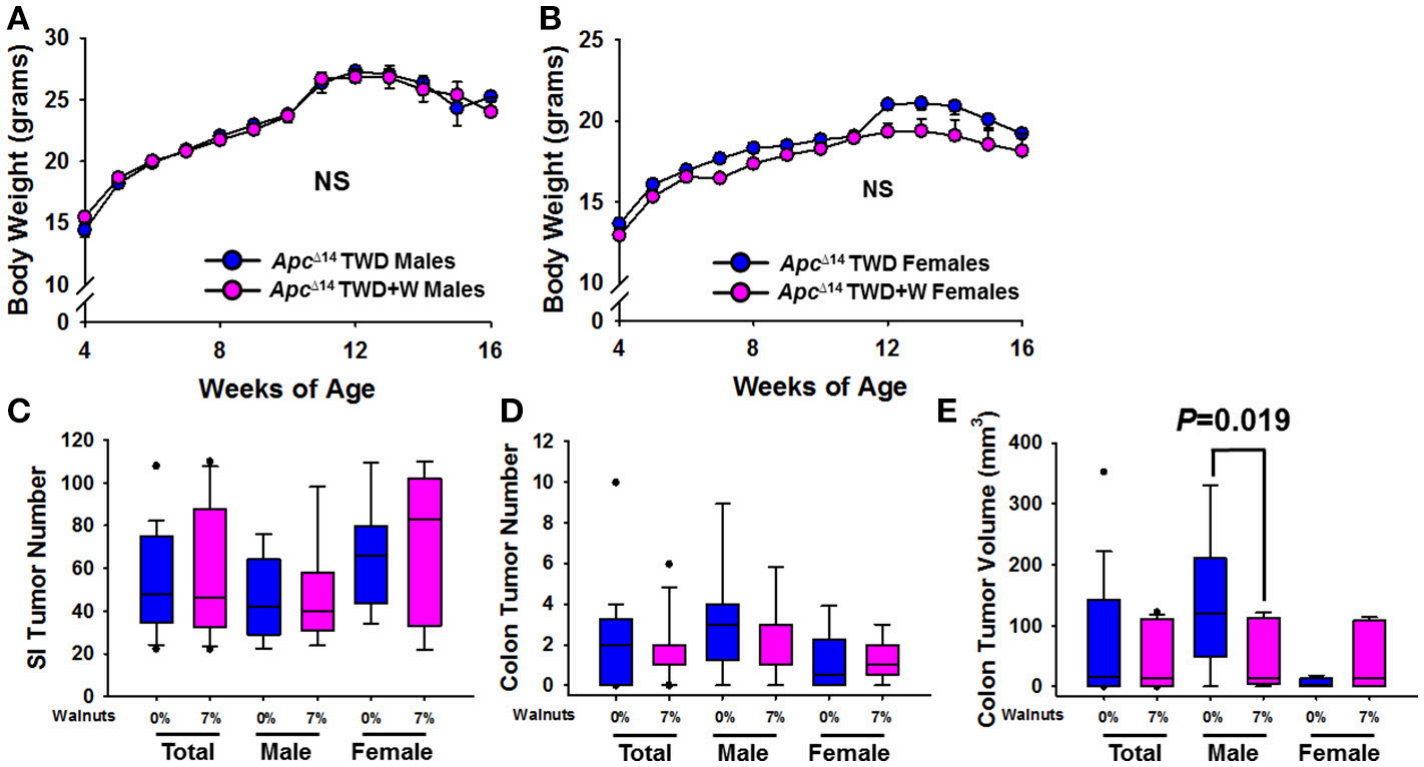
Effect of diet and walnut consumption on intestinal tumorigenesis in *Apc*^Δ14/+^ male and female mice. Tumorigenesis was studied in a second mouse model of intestinal tumorigenesis (*Apc*^Δ14/+^). Male (Con TWD *n* = 12, TWD+7% Walnut *n* = 11) and female (Con TWD *n* = 10, TWD+7% Walnut *n* = 9) mice were provided either a control TWD or TWD supplemented with 7% walnuts, a dose range in which we have previously determined to confer optimal protection against tumorigenesis. **(A,B)** There was no effect of walnuts on weight gain with TWD in either male or female mice, further supporting that walnuts do not alter energy balance in mice. **(C,D)** Tumor latency and multiplicity is more severe in the *Apc*^Δ14/+^ mouse than in Apc1638 animals, but tumor multiplicity was not significantly different between groups in either sex. **(E)** When average tumor volume was determined in the colon, size was significantly reduced only in male mice (*P* = 0.019). Lines and symbols represent mean ± SE. Box plots indicate median values and first to third quartile range, while lines indicate minimum and maximum range; symbols indicate outliers. NS, Not significant.

In order to further confirm the protective effects of walnuts on tumorigenesis within the context of an unhealthy diet, we next performed a study in *Apc*^Δ14/+^ male and female mice, using an established TWD paradigm, which we have previously used to demonstrate a protective effect of walnuts in the AOM colon cancer model (24). Inclusion of walnuts in the TWD had no effect on weight gain in either males or females (Figures 6A,B), which is consistent with other data on weight gain. Further, no effect on tumor multiplicity was observed in the small intestine for either sex (Figure 6C), but a slight numerical reduction in colon tumor number was observed in males (Figure 6D), which was accompanied by a significant reduction in colon tumor volume in males only (Figure 6E; *P* = 0.019). However, this was not associated with changes in proliferation markers, including Ki67 and β catenin staining by IHC (Supplementary Figure 2).

**Figure 7:**
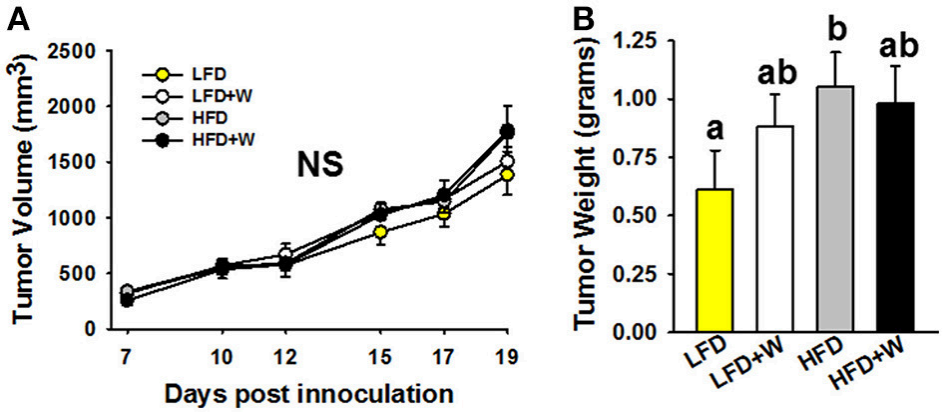
Effect of diet and walnut consumption on MC38 colon cancer cell proliferation *in vivo***. (A,B)** Based upon tumor volumes collected over 19 days post-inoculation, no significant difference in growth was observed. However, when tumor weights were collected at necropsy, there was a tendency for increased size in LFD+W mice while this was significant for the HFD only group, as compared to LFD controls. Bars represent mean ± SE. NS, Not significant. Different letters denote a significant difference between groups, *P* ≤ 0.05.

Finally, we evaluated the effect of walnuts on colon cancer cell progression in male C57BL/6J mice. Following the injection of tumor cells, palpable tumors were detected by day 7 and tumor volume was monitored for 19 days after injection. Tumor growth, based upon volume measurements, tended to be greater in HFD and HFD+W mice by day 19, as compared to LFD groups, but did not reach statistical significance (Figure 7A). However, tumor weight was significantly greater in HFD mice, as compared to LFD controls (*P* < 0.05), while tumor weight tended to be greater in LFD+W mice, as compared to their respective controls (Figure 7B). Furthermore, in contrast to the protective effects of walnuts conferred on obesity-accelerated tumorigenesis, growth was not mitigated in HFD+W mice, suggesting walnuts do not confer protection in lean or obese animals against MC38 cancer cell progression *in vivo*.

## Discussion

It has been proposed that cancer development is an inevitable consequence of multiple stem cell divisions and stochastic processes that provoke DNA damage, increasing the probability for tumorigenesis (43). However, an extensive body of evidence has strongly implicated environmental and other lifestyle factors as significant modulators of cancer risk, including colon cancer (44). Obesity has proven to be particularly hazardous for cancer risk at many sites, as well as accelerated disease progression and related mortality (2, 3). Such evidence is alarming given that more than two-thirds of US adults are now overweight or obese (45, 46), and obesity rates are expected to remain a major health concern despite public health efforts to reverse this trend. In fact, based upon current estimates of obesity and its probable health consequences, it is estimated that 65 million more US adults will be obese by 2030, resulting in 492,000–669,000 new cancer cases in the US alone (47). Therefore, simple and cost-effective strategies are urgently needed that can break the obesity-cancer link.

Over the past several decades, nut consumption generally, and dietary walnuts specifically, have proven beneficial for aspects of human health, including improved lipid profiles (16, 48, 49) and endothelial function, while epidemiologic studies have linked walnut intake with reduced incidence of type 2 diabetes, cognitive decline, and cardiovascular disease-related mortality (13–16). More recently, several preclinical and *in vitro* studies have uncovered beneficial effects of walnuts and its components on cancer risk (17), particularly in colon cancer models (24, 26, 27, 50–52). Given that walnuts are enriched with a complex composition of bioactives that could interfere with the tumor-promoting effects of the obese environment, we reasoned that they could prove particularly efficacious in mitigating obesity-related cancer risk. Consistent with our hypothesis, we show in two independent *Apc* models of intestinal cancer, that walnuts confer protective effects against tumor incidence and growth, and that these effects appear to be most prominent under metabolic duress, as no such effect was observed when animals were provided a LFD. However, these benefits appear to be limited to tumor initiation and promotion, as we failed to observe any protective effect of walnuts on MC38 colon cancer cell growth rates *in vivo*, either under low or high fat dietary conditions. The latter results are in contrast to a prior study reporting an inhibitory effect of walnut consumption on HT-29 colon cancer cell growth in inoculated mice (26), which was attributed to decreased angiogenesis and altered miRNA and fatty acid profiles in the tumor cells (27). Thus, while the evidence linking walnuts to reduced tumorigenesis appears to be increasingly robust, their ability to slow colon cancer progression is less clear and would benefit not only from additional studies, but also from better models that more closely recapitulate the pathophysiology of human metastatic disease.

A second important observation from these studies is that the benefits of walnuts on tumor growth appear to be sexually dimorphic, with preferential protection conferred in male mice, which is in agreement with a previous study on walnuts and colon cancer (24). Sex differences are now well-recognized to play a major role in responses to various pharmacologic interventions on lifespan and healthspan (53), as well as in the etiology of many diseases, including colon cancer. Indeed, a meta-analysis of prospective studies determined that an elevated body mass index (BMI>30) increases the risk for colon cancer by 30–70% in men, but less so in women (4, 54). This sex difference was also apparent in a large prospective study (European Prospective Investigation into Cancer and Nutrition; EPIC) (55), which found that the risk of colon cancer was 55% greater in the highest quintile of BMI, as compared to the lowest quintile in men, but no association was observed in women (5). Interestingly, the risk of colon cancer in women increases after menopause and is modulated by hormone replacement, highlighting an important protective role for sex hormones in the pathophysiology of this disease (56). Thus, the presumably high levels of female sex hormones during tumor development in these mice may have obscured any protective effect of walnuts on tumorigenesis. Future studies interrogating the effect of walnuts and other interventions on intestinal cancer should account for hormonal status as a potential modifier of the response.

Although the ability of walnuts to mitigate intestinal tumors, particularly in obesity, is now well demonstrated, the mechanism(s) underlying this protection are not entirely clear. Metabolic dysfunction, including insulin resistance, along with the chronic low-grade pro-inflammatory state typical of obesity, are commonly cited as major drivers of obesity-related cancers (57), and we reasoned that walnuts could potentially interfere with one or more of these processes. However, we were unable to detect such effects of walnuts on any metabolic parameters tested, although in HFD+W we did observe a marked reduction in plasma CCL5 levels, and this cytokine has been linked to colon cancer growth and progression (58–60). Meanwhile, consistent with many observations, adipose inflammation was markedly increased in HFD-fed mice, irrespective of walnut intake, suggesting that a reduction in adipose inflammation is not a mechanism linking walnuts to reduced tumorigenesis in these mice.

Beyond potential systemic mediators of the cancer-preventative effects of walnuts, the proximity of walnut digestion and absorption to the gut suggests a high probability that direct inhibition of tumor growth or other alterations to the gut environment are involved in the attenuation of tumor growth by dietary walnuts. Indeed, several studies have demonstrated that individual components contained in walnuts, including α-linolenic acid (ALA), γ-tocopherol, carotenoids, and polyphenols, all harbor the ability to retard cancer cell growth *in vitro* and *in vivo* (17). Walnuts also contain significant amounts of fiber, which has been associated with a modest reduction in colorectal cancer risk in some reports (61–63). More recently, two rodent studies have demonstrated that dietary walnuts can significantly and favorably modulate the composition of the gut microbiome (24, 28), including an increase in microbial diversity and enrichment for more probiotic-type bacteria including *Firmicutes, Lactobacillus, Ruminococcaceae*, and *Roseburia*, and a reduction *Bacteroides* and *Anaerotruncus*, and these shifts in structure have been associated with less colon cancer risk. Further, if alterations to the gut microbiome are indeed involved in retarding tumor development and growth, this may explain in part why walnuts failed to slow colon cancer cell progression in the xenograft model, in which MC38 cells were implanted into the animal's flank, entirely removed from the normal gut microenvironment.

A final consideration relates to how the experimental diets were designed, which involved rigorously matching for calories and macronutrient composition. Achieving this level of control in the HFD formulas required among other adjustments, reducing saturated fat content in the form of lard by ~25% in the HFD+W formulation, to account for the fat content in walnuts. Thus, we cannot completely rule out that this modest displacement in fat and/or other nutrient sources could have contributed to some of the protective effects in the HFD+W group, though this seems unlikely, as lard content in this diet still remained exceedingly high, as compared to the LFD formula. Furthermore, while red meat intake is generally associated with colon cancer (64, 65), the association between saturated fat *per se* and colon cancer risk in humans is somewhat controversial (66, 67), while evidence in rodents supports both increased (68) and decreased risk for colon cancer risk by saturated fat (69).

Since the discovery of specific ISC populations, including Lgr5+, Bmi1+, or Lrig1+ ISCs (70–72), and their ability to serve as the origin of intestinal tumor development, much interest has been focused on their function in the context of diet and aging. Obesity *per se* has also been shown to induce hypertrophy of the intestinal mucosa (8), changes to the colonic epigenomic landscape that favor growth (10), increased ISC proliferation, stemness, and accelerated tumor development in *Apc*-deficient Lgr5+-ISCs (9). However, ISC function has also been shown to be impaired in aging (73), while the age-delaying strategies, rapamycin and caloric restriction, boost ISC function (42). Here, we observed impaired ISC function in young mice on a HFD, while ISC proliferation, as determined by organoid formation, was markedly maintained by walnut supplementation. While the exact functional relevance of this effect on ISC function will require further study, it provides an intriguing example of how walnuts are able to preserve a key aspect of intestinal homeostasis in obesity.

As previously mentioned, a HFD was previously shown to promote ISC proliferation and stemness, effects that were proposed to correlate with the increased proliferative potential of gut stem-cell derived cancers in obesity (9). However, we observed a contrasting result, whereby obesity impaired ISC function that was preserved by walnuts. While a definitive explanation of these disparate results is not entirely clear, an important distinction between our approach (36) and others (9), is that our HFD-fed animals are compared to a carefully-matched, low fat purified diet, rather than a poorly-defined chow-based formula. The contrasting compositions of purified vs. chow based formulations, and the ability of multiple individual components in chow diet to impact intestinal function and tumorigenesis, independent of calories, has been extensively discussed elsewhere (36, 74).

In summary, these data demonstrate that walnuts confer significant protection against intestinal tumorigenesis and growth in the context of obesity and a high-calorie diet. Previous studies have linked various mechanisms to the protective effects of walnuts in colon cancer, including alterations to the microbiome, and we further noted reductions in circulating CCL5 in HFD mice supplemented with walnuts, though changes in tissue inflammation or metabolic signaling did not seem to be impacted by walnuts. Furthermore, these data demonstrate that even prior to tumor initiation, walnuts preserve aspects of intestinal homeostasis by mitigating the impairment in ISC function imposed by a HFD. Thus, these findings, along with prior observations, strongly suggest that walnuts can serve as a potentially-effective dietary strategy to break the obesity-colon cancer link, a possibility which should be further explored in human trials.

## Ethics statement

All experimental animals were housed and treated in accordance with protocols reviewed and approved by the Institute for Animal Care and Use Committee at the Albert Einstein College of Medicine (Protocols #20150103) and the Animal Care Committee at the University of Connecticut Health Center, respectively. Data were rigorously collected by several team members, repeated when necessary, and were summarized, reviewed, and approved by all authors without bias, fabrication or data manipulation.

## Author contributions

DH and DR contributed to the conception and design of the study, data analysis and interpretation, and wrote and edited the manuscript. FG and MN designed experiments, developed methodologies, performed experiments, and wrote and edited the manuscript. TT and AN contributed to experimental design, development of methodology, performed the experiments and edited the manuscript.

### Conflict of interest statement

The authors declare that the research was conducted in the absence of any commercial or financial relationships that could be construed as a potential conflict of interest.
