# Supplementary material for: Dietary Walnuts Protect Against Obesity-Driven Intestinal Stem Cell Decline and Tumorigenesis
Source: Front Nutr. 2018 May 31;5:37. doi: 10.3389/fnut.2018.00037 (PMC5990619; doi:10.3389/fnut.2018.00037)
Supplement: Supplementary file 1 [file Image_1.pdf]

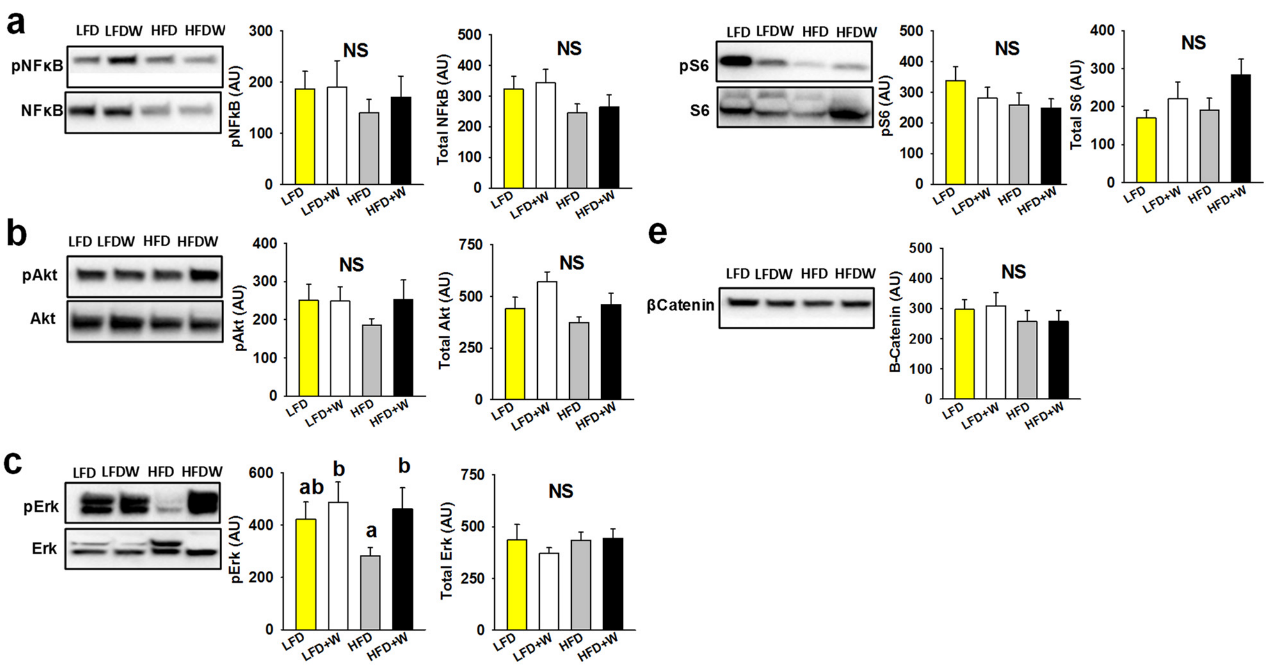

Supplementary Figure 1. Effect of diet and walnut consumption on metabolic signaling pathways in colon. (**a-b**) In colon, no significant effect was observed for pNFκB (p65) or Akt signaling (*n*=8 per group). (**c**) In contrast to the jejunum, HFD significantly decreased pErk, which was prevented in HFD+W mice. (**d-e**) There were no differences among groups for pS6 or β-catenin levels among groups. Bars represent mean±SE. NS=Not significant. Different letters denote a significant difference between groups, *P*≤0.05.
