# Supplementary material for: Dietary Walnuts Protect Against Obesity-Driven Intestinal Stem Cell Decline and Tumorigenesis
Source: Front Nutr. 2018 May 31;5:37. doi: 10.3389/fnut.2018.00037 (PMC5990619; doi:10.3389/fnut.2018.00037)
Supplement: Supplementary file 2 [file Image_2.pdf]

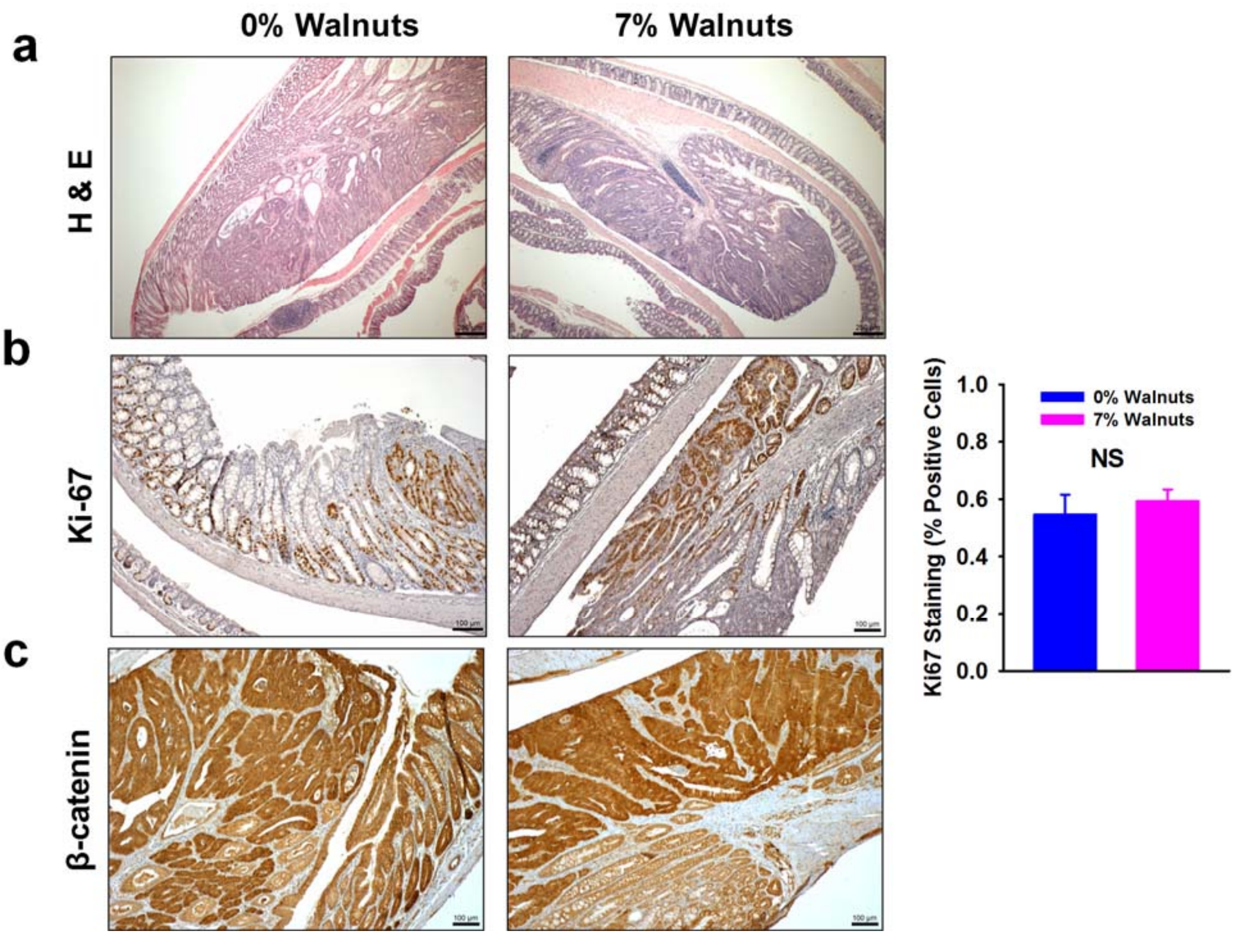

Supplementary Figure 2. Effect of walnut consumption on growth and proliferation pathways in colon. (**a**) H&E staining was performed on Con male mice fed a 0% Walnut TWD (*n*=3) 0% and 7% Walnut-fed male mice (*n*=4). (**b-c**) In colon, no significant effect was observed on Ki67 positive cells nor was any effect observed on β-catenin staining in male mice. Bars represent mean±SE. NS=Not significant.
