# Supplementary material for: Dietary Walnuts Protect Against Obesity-Driven Intestinal Stem Cell Decline and Tumorigenesis
Source: Front Nutr. 2018 May 31;5:37. doi: 10.3389/fnut.2018.00037 (PMC5990619; doi:10.3389/fnut.2018.00037)
Supplement: Supplementary file 3 [file Table_1.PDF]

Supplementary Table 1. qRT-PCR Primer Sequences

Gene	Sequence
ZO-1	For 5'-CCACCTCTGTCCAGCTCTTC<br>Rev 5'-CACCGGAGTGATGGTTTTCT
JAM-A	For 5'-GTTCCCATTGGAGTTGCTGT<br>Rev 5'-GGGCACAGGGGAGAAATCG
TNF-α	For 5'-CCCTCACACTCAGATCATCTTCT<br>Rev 5'-GCTACGACGTGGGCTACAG
IL-1β	For 5'-GAAATGCCACCTTTTGACAGTG<br>Rev 5'-TGGATGCTCTCATCAGGACAG
Occludin	For 5'-CCTCCAATGGCAAAGTGAAT<br>Rev 5'-CTCCCCACCTGTCGTGTAGT
Claudin	For 5'-AAGGTGCTGAGGGTAGA<br>Rev 5'-TTGAGCATTCAAAGCACAGG
18S	For 5'-TTGACGGAAGGGCACCACCAG<br>Rev 5'-GCACCACCACCCACGGAATCG
